# Robust ParB Binding to Half-*parS* Sites in *Pseudomonas aeruginosa*—A Mechanism for Retaining ParB on the Nucleoid?

**DOI:** 10.3390/ijms241512517

**Published:** 2023-08-07

**Authors:** Adam Kawalek, Aneta Agnieszka Bartosik, Grazyna Jagura-Burdzy

**Affiliations:** Laboratory of DNA Segregation and Life Cycle of Proteobacteria, Institute of Biochemistry and Biophysics, Polish Academy of Sciences, 02-106 Warsaw, Poland

**Keywords:** *Pseudomonas aeruginosa*, chromosome segregation, partitioning proteins, ParB distribution, half-*parS*s

## Abstract

Chromosome segregation in *Pseudomonas aeruginosa* is assisted by the tripartite ParAB–*parS* system, composed of an ATPase (ParA), a DNA-binding protein (ParB) and its target *parS* sequence(s). ParB forms a nucleoprotein complex around four *parS*s (*parS1–parS4*) that overlaps *oriC* and facilitates relocation of newly synthesized ori domains inside the cells by ParA. Remarkably, ParB of *P. aeruginosa* also binds to numerous heptanucleotides (half-*parS*s) scattered in the genome. Here, using chromatin immunoprecipitation-sequencing (ChIP-seq), we analyzed patterns of ParB genome occupancy in cells growing under conditions of coupling or uncoupling between replication and cell division processes. Interestingly, a dissipation of ParB–*parS* complexes and a shift of ParB to half-*parS*s were observed during the transition from the exponential to stationary phase of growth on rich medium, suggesting the role of half-*parS*s in retaining ParB on the nucleoid within non-dividing *P. aeruginosa* cells. The ChIP-seq analysis of strains expressing ParB variants unable to dislocate from *parS*s showed that the ParB spreading ability is not required for ParB binding to half-*parS*s. Finally, a *P. aeruginosa* strain with mutated 25 half-*parS*s of the highest affinity towards ParB was constructed and analyzed. It showed altered ParB coverage of the *oriC* region and moderate changes in gene expression. Overall, this study characterizes a novel aspect of conserved bacterial chromosome segregation machinery.

## 1. Introduction

The accurate distribution of genetic material between progeny cells is fundamental for the continuity of species. In Eukaryotes, the consecutive steps of this process are spatiotemporally separated, whereas in Prokaryotes they proceed in the same confined space and overlap in time, which ultimately requires mechanisms for coordination of the genome duplication and segregation with cell growth and division [1,2,3]. Recent studies revealed that genome organization and segregation in bacterial cells involve universal as well as species-specific mechanisms [2,4,5,6,7,8]. The majority of chromosomal and extrachromosomal DNA molecules in bacteria encode active partitioning systems, relying on three elements: two partner proteins, an NTPase (ParA) and a DNA binding protein (ParB), as well as palindromic centromere-like sequences, *parS*s, located in the chromosomes in the vicinity of the origin of replication—*oriC* [9]. ParB binds *parS*s and spreads on the DNA surrounding these sites to form large nucleoprotein complexes [10,11]. ParB molecules are capable of binding and hydrolyzing cytidine triphosphate (CTP), the processes that engage amino acid residues from the conserved arginine rich patch in the N-terminal part of the protein and drive switching between open and closed (ring-like, DNA-associated) conformations of ParB molecules [12,13,14,15,16]. The ParB–*parS* complexes are ultimately distributed to the dedicated cell compartments by the ParB cognate partner, ParA [1,17,18]. The Par proteins collaborate with various cell machinery, e.g., the replisome, DNA topology determinants, the divisome and polar hubs, to precisely coordinate the DNA segregation process with the cell cycle. Notably, the chromosome orientation and compaction can be significantly modified in response to the changing environmental conditions, e.g., starvation during sporulation in *Bacillus subtilis* or *Streptomyces*, suggesting flexibility of molecular mechanisms involved in the process [19,20,21,22,23,24].

In *Pseudomonas aeruginosa* (*Pae*), an opportunistic human pathogen, the chromosome choreography, spatial organization and segregation were investigated under various conditions [25,26,27]. The deletion of the *parA* or *parB* gene in *Pae* was not lethal; however, the growth rate, cell size, colony morphology, motility and number of anucleate cells produced during cell division were altered in the mutants in comparison to the parental strain [28,29]. *Pae* ParB was shown to bind with the highest affinity to four palindromic *parS*s (*parS1* to *parS4*) situated in the vicinity of *oriC*, and at least one of them was sufficient to support chromosome segregation [30,31]. Interestingly, in vitro and in vivo experiments showed ParB binding to at least five other *parS*-like sequences containing two nucleotide substitutions [27,30,32]. Moreover, a genome-wide analysis of ParB binding in *Pae* using chromatin immunoprecipitation and sequencing (ChIP-seq) indicated binding to heptanucleotide motifs corresponding to one arm of the *parS* motif (half-*parS* sites) [32]. In cells of the exponentially growing strain PAO1161 (WT), a few hundred half-*parS* sites were detected as ParB-enriched; however, this number increased to around a thousand when ParB amount was not limited (overproduction) or the *parS1–parS4* sites were inactivated. This observation strongly implies that the pattern of distribution of ParB protein in the genome might change under certain circumstances, e.g., the level of the available ParB molecules in the cells. The presence of multiple binding sites [32], global changes in the expression of genes observed in *Pae parB* deficient mutant [33] and ParB interactions with multiple partners [34] suggested the role of ParB extending beyond *oriC* region segregation.

Here, we investigated ParB–*parS*s and ParB–half-*parS* interactions in *Pae* cells growing with different division times. In fast-growing cells, the cell division and replication cycles are uncoupled [35]. The division time is shorter than needed for chromosome duplication, as cells begin another round of replication before the previous cell division has been completed. In contrast, in bacterial cells growing slowly, e.g., due to the environmental factors, the cell division is strictly coupled with the chromosome duplication [1]. Such coupling of both processes also occurs when fast-growing cells in a batch culture approach the stationary phase of growth [26,36]. Here, we systematically investigated the ParB binding pattern to the genome in the *Pae* WT strain and *parS1–4* mutant grown with varying cell division times: in rich medium (LB) or in mineral medium (M9 + glucose), at two temperatures (30 °C, 37 °C), at exponential and stationary stages of culture growth. Genomic distribution of three ParB variants, deficient in spreading, was also analyzed using ChIP-seq. Furthermore, we constructed a *Pae* mutant with 25 mutated half-*parS*s, around which the repeatedly highest ParB ChIP-seq peaks were formed in the WT strain, to investigate the relationship between ParB binding to these sites, ParB distribution on the genome and gene expression.

## 2. Results

### 2.1. Distribution of ParB on Genome in Cells from Exponential and Stationary Phases of Cultures under Conditions of Varying Cell Division Time

**Figure 1:**
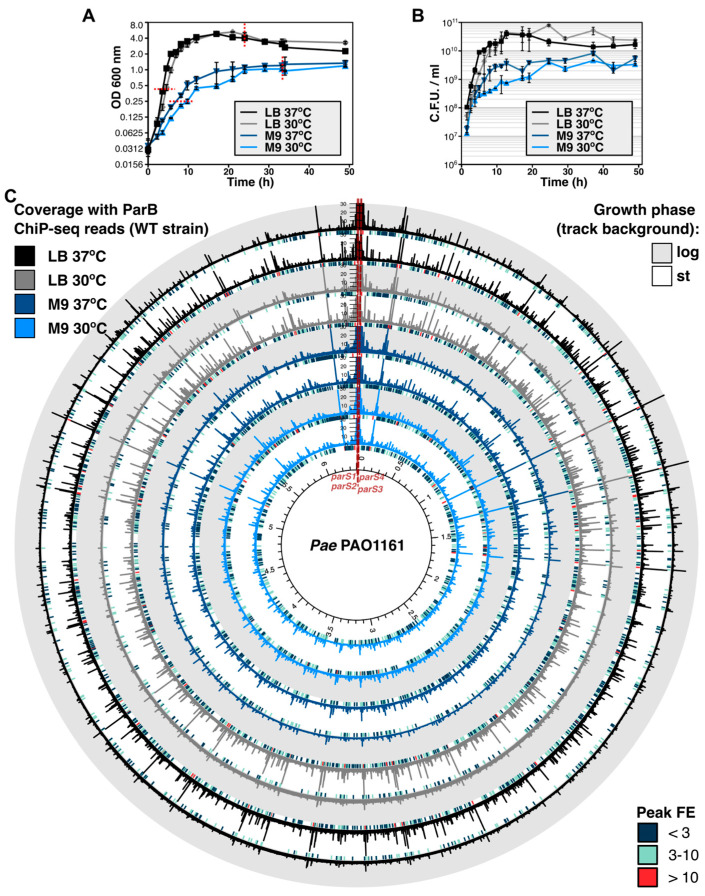
ParB binding to the *P. aeruginosa* chromosome is dependent on the growth conditions. Growth of *Pae* PAO1161 (WT) strain in LB or M9 medium +0.5% glucose at 30 °C or 37 °C presented as (**A**) mean optical density at 600 nm ± SD from three cultures and (**B**) mean number of colony-forming units (CFUs) per mL ± SD assayed for three cultures. (**C**) ChIP-seq analysis of ParB binding to genome assayed using WT strain cells from cultures in LB or M9 medium, grown at 30 or 37 °C, at two time points representing exponential and stationary phases of culture growth (indicated on (**A**) with red lines). Track data represent normalized coverage of the genome with ChIP-seq reads (binned at 200 bp and averaged from two ChIP-seq replicates). Color of coverage track represents different growth conditions, whereas track background indicates the phase of culture growth. Track height was set at 10% of max. coverage signal of the outer track. Short bars below each track represent the distribution of detected ChIP-seq peaks for these samples, colored according to the peak fold enrichment (FE).

To assess the effect of growth conditions on distribution of ParB on the bacterial chromosome, we performed a set of ParB ChIP-seq experiments using cells of PAO1161 (WT) strain grown on rich medium (LB) as well as minimal M9 medium with glucose at two different temperatures (37 °C and 30 °C) from the exponential and stationary phases of culture growth (Figure 1A). The division time of exponentially growing culture on the rich medium was 33 ± 5 min at 37 °C and 44 ± 5 min at 30 °C, whereas it was extended to 97 ± 11 min at 37 °C and 163 ± 25 min at 30 °C, respectively, for cultures on minimal medium (Figure 1B). Additionally, PAO1161 *parS1*–*4* strain with inactive four *parS* sites was used to analyze the ParB distribution on the chromosome in the absence of ParB–*parS* complex [32]. The Western blot analysis did show the presence of ParB in the input samples used for ChIP-seq experiments, for all conditions tested (Figure S1). After the ChIP-seq procedure, reads were mapped to the PAO1161 reference genome [37], and peaks were called together for the two biological replicates, using the data obtained for Δ*parB* as a control.

The coverage of the genome with ChIP-seq reads and consequently the number of ParB peaks varied most significantly between the samples from the exponentially growing versus stationary cultures on the rich medium, with the coverage with reads and peak fold enrichment (FE) for the majority of peaks elevated in samples derived from stationary cultures (WT log 30 °C and WT log 37 °C, Figure 1C). No major differences in the number of peaks and their FE were observed between the cultures grown on the minimal medium (four inner tracks, Figure 1C). Similar analysis of data obtained for *parS1*–*4* strain showed that, in contrast to the WT strain, an increase in the number of ParB-enriched regions was observed for samples derived from the logarithmic phase of the growth at 37 °C on LB in comparison to the stationary phase of the same culture (Figure S2). Otherwise, the distribution of ParB peaks and their FE were not significantly affected by the tested growth conditions in this strain (discussed below).

**Figure 2:**
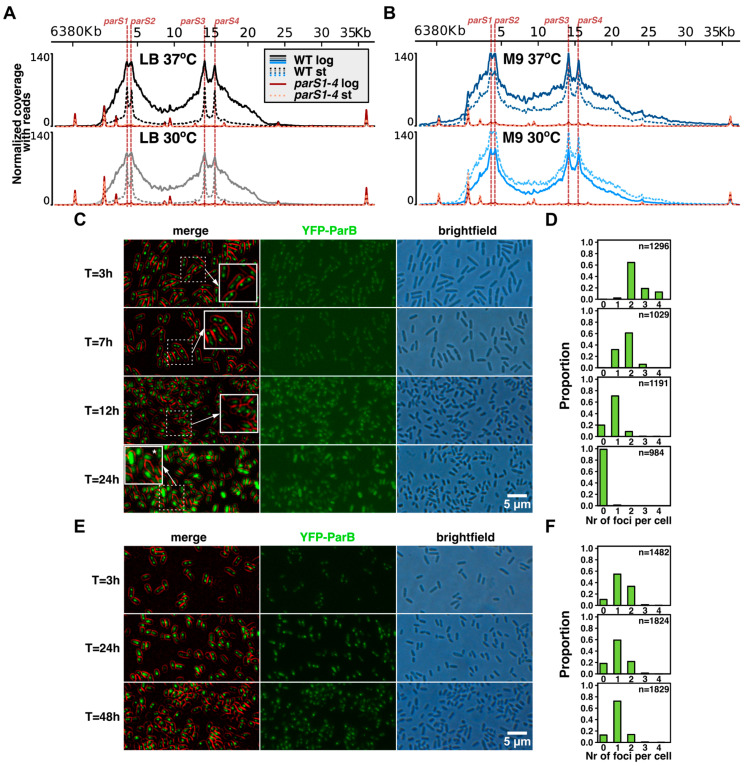
Formation of ParB–*parS* complexes in *Pae* PAO1161 cells depends on the growth conditions. (**A**,**B**) Coverage of the genome regions encompassing the *parS1*–*parS4* in the PAO1161 genome in the ChIP-seq samples for the indicated strains/culture conditions. The histograms show normalized coverage with reads (averaged for two biological replicates). (**C**–**F**) Microscopic analysis of PAO1161 YFP–ParB producing cells grown in (**C**) LB medium at 37 °C or (**E**) M9 medium with 0.5% glucose at 37 °C at different time points of culture growth. On the merged images, the fluorescence signal was enhanced, and the cell contour was false-colored in red. Marked areas were copied and enlarged (**C**), and the asterisk at the 24 h panel indicates a rare cell with foci in this conditions. (**D**,**F**) Histograms showing distribution of the number of YFP–ParB foci per cell, detected in fluorescence images using MicrobeJ ver 5.13m.

Previous analyses of the region around *oriC*, harboring the *parS1*–*parS4* cluster, in ChIP-seq samples of the WT strain growing exponentially showed the presence of a broad (>30 kb) ParB-enriched region, being the hallmark of the ParB nucleoprotein complex formation by spreading around *parS*s [31,32]. Here, the presence of the ParB complex in the *oriC* region was also evident in the fast-dividing WT cells growing exponentially on rich medium (uncoupled replication and cell division), whereas enhanced coverage of regions around *parS*s was not observed in samples derived from stationary cultures, although in these samples ParB binding to the individual *parS*s was still observed (Figure 2A). Notably, when cells were grown in minimal medium under conditions of coupling between replication and cell division, the ParB–*parS* nucleoprotein complex was present in cells at both analyzed time points (Figure 2B), representing logarithmic and stationary phases of culture growth (as concluded by analysis of changes in the amount of colony-forming units per ml of culture, Figure 1B).

To track the ParB–*parS1*–*parS4* complex formation in *Pae* cells under different culturing conditions, we applied fluorescence microscopy using YFP–ParB fusion protein as described previously [34]. The *tac* promoter used here was shown to be leaky in *Pae* cells [38], and the plasmid used (a pBBR derivative) was shown to be extremely stable in *Pseudomonas* [39] (and our unpublished data); hence, no inducer or chloramphenicol were added to the cultures to match the culturing conditions with those used for ChIP-seq. The YFP–ParB foci presence was tracked in time in WT cultures grown in LB and M9 + glucose at 37 °C (Figure 2C–F). The number of detected ParB foci per cell changed drastically between exponential and stationary cultures grown on LB (Figure 2D). Cells growing exponentially had mostly two to four foci per cell; the majority of cells from the transition phase had only one focus per cell, whereas the YFP–ParB signal was mostly dispersed in the cells grown for 24 h (Figure 2D). Similar analysis of cells grown in M9 showed that YFP–ParB foci were present even after 48 h of culturing. Overall, the ChIP-seq and microscopy results indicate that ParB–*parS* complexes are present in *Pae* cells, even in the populations of non-dividing cells, and are only being disassembled in cells from the high-density cultures, at late stages of growth.

### 2.2. Growth Conditions Affect the Extent of ParB Binding to Half-parSs but Not the Individual Half-parS Preference

**Figure 3:**
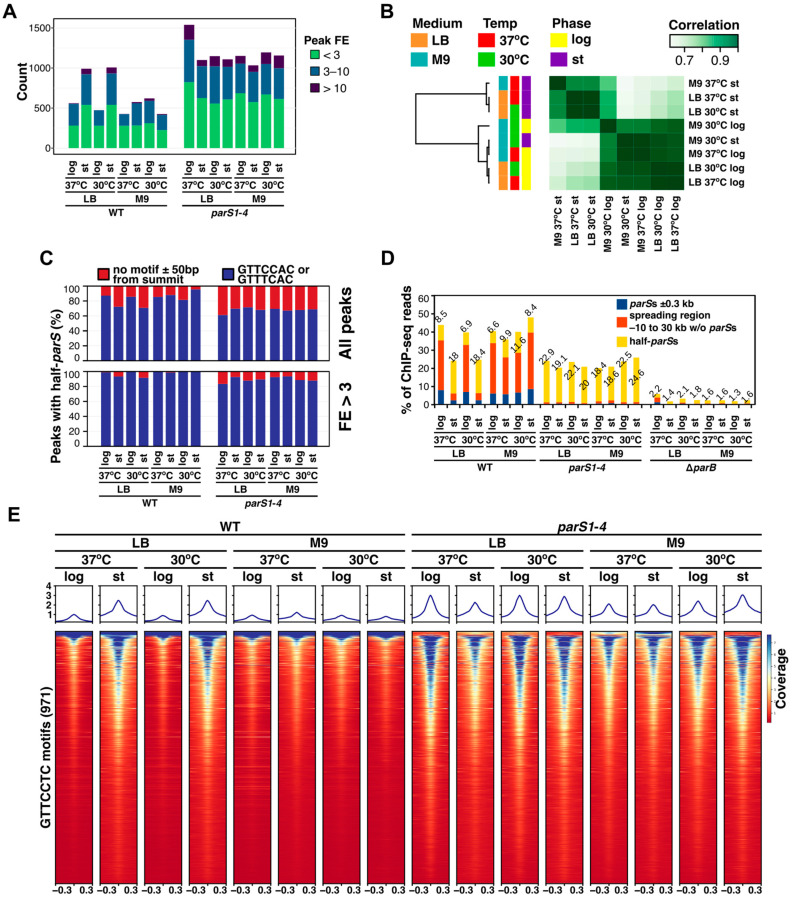
Redistribution of ParB from complex around *parS*s to half-*parS* sites with changes in growth conditions. (**A**) Number of ParB ChIP-seq peaks for samples derived from the indicated strain and growth conditions, categorized according to their fold enrichment (FE). (**B**) Correlation heatmap between different ChIP-seq peak sets, based on occupancy and MACS2 scores. (**C**) Percentage of ChIP-seq peaks containing GTTCCAC or GTTTCAC (half-*parS*) motifs ± 50 bp from the summit. The analysis was performed for all peaks (upper panel) and for peaks with FE > 3 (bottom panel). (**D**) Percentage of ChIP-seq reads mapping to genome regions encompassing *parS1–4* or half-*parS*s in different samples. Reads mapping to *parS*s ± 300 bp, ParB spreading zone without *parS*s and regions with half-*parS*s (GTTCCAC and GTTTCAC ± 300 bp, altogether 11.6% of the genome) were counted, and background (based on the amount of reads mapping to the remaining genome regions) was subtracted. Data for ChIP-seq replicates were averaged. (**E**) Read coverage around GTTCCAC motifs in the PAO1161 genome in ParB ChIP-seq samples. Individual lines in the heatmap represent normalized read counts for regions ±300 bp around a motif. The data were sorted in descending order of median coverage value and represent averaged value for two biological replicates. Plots indicate median coverage over the analyzed regions.

In addition to ParB binding to *parS*s, numerous ParB-enriched loci were observed in the ChIP-seq experiments (Figure 1C). The number of ParB ChIP-seq peaks in WT strain samples varied between 400 and 600 except for samples derived from the stationary cultures grown on rich medium, which showed around 1000 peaks (Figure 3A, Table S1). The number of ParB peaks in *parS1–4* mutant samples was around 1000 for all growth conditions/phases of growth, with the exception of the samples derived from cells grown at 37 °C in rich medium in the logarithmic phase of growth in which more ParB occupied sites were identified (Figure 3A). Comparison of occupancy maps (i.e., comparison of genome positions identified as peak ranges along with peak caller scores) showed a high correlation for all the analyzed peak sets (Figure 3B). The discriminating factor, grouping the samples, was the peak number, which indicates that the same sites were detected as ParB-enriched in the majority of ChIP-seq samples. The analysis of detected ParB ChIP-seq peak sequences showed that, for all samples, more than 80% of peaks had summits in proximity of GTTCCAC or GTTTCAC half-*parS* motifs (Figure 3C), sequences identified previously as the heptanucleotide variants most preferentially bound by ParB [32]. This percentage was even higher among peaks with fold enrichment >3, confirming that these half-*parS* motifs are the main variants bound by ParB in *Pae* irrespective of the culturing conditions.

To analyze the distribution of ParB between genome regions with *parS*s and those with half-*parS*s sites, the number of ChIP-seq reads mapping to these regions was counted (Figure 3D). The region spanning −10 kb to 30 kb from the starting position of the assembled genome was designated as the ParB spreading zone in this analysis, as the majority of ParB was gathered in this region (Figure 2A,B and [31,32]). Since the half-*parS* sites containing regions ±300 bp spanned approx. 11% of the genome, the theoretical background was subtracted from all read counts, to only consider the ‘true’ ParB ChIP-seq signal. In samples derived from WT fast-growing cells in LB at 37 °C, about 35% of ChIP-seq reads mapped to the ParB spreading zone with roughly 8% of it covering 2.4 kb of the genome around *parS1* to *parS4* sites (Figure 3D). About 9% of the reads mapped to half-*parS*s. In the cells from corresponding cultures at the stationary phase of growth, the amount of ParB recovered from the spreading zone dropped to 5.8% with 2% bound to the individual *parS1* to *parS4* sites, and the amount bound to half-*parS*s doubled to 18% (Figure 3D). Identical change of ParB distribution was observed in cells grown in LB at 30 °C, whereas no changes in the ParB distribution in the various phases of culture growth were observed in WT slow-growing cells in the mineral medium. These data suggest that ParB not involved in the formation of ParB–*parS* complex may be redistributed to half-*parS* sites. In the samples derived from mutant cells deprived of the *parS1–parS4* sites, around 20% of ParB ChIP-seq reads mapped to half-*parS*s under all growth conditions (Figure 3D). When a similar analysis was conducted for unrelated DNA binding proteins with multiple binding sites in the genome, *Pae* PA2121 (a LysR type transcriptional regulator, 765 peaks [40]) and PA3458 (a MarR transcriptional regulator, 1183 peaks [41]) the percentage of reads mapping to the ChIP-seq peaks was 22% and 43%, respectively, indicating that the values obtained for ParB resemble those obtained for regulators with multiple binding sites in the genome.

**Figure 4:**
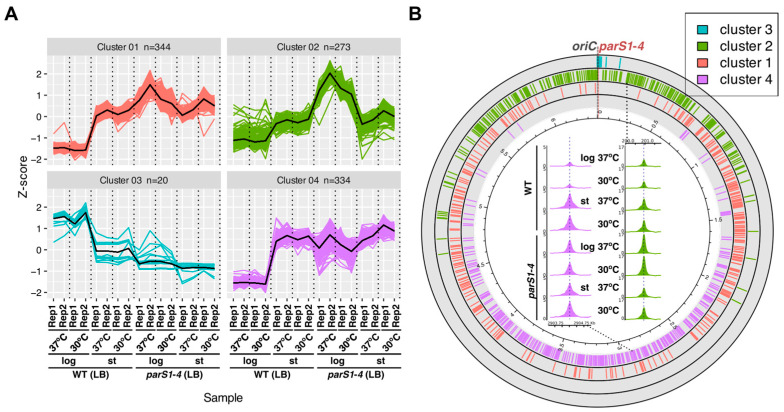
The preference of ParB binding to individual half-*parS* motifs is unaffected in cells grown in different media, at the tested temperatures and does not depend on the phase of growth. Coverage data for all 971 GTTCCAC motifs present in the PAO1161 genome were extracted from ChIP-seq data, normalized (Z-score) and subjected to k-means clustering. (**A**) ChIP-seq read coverage profiles for motifs in each cluster. The *y*-axis represents the difference (in standard deviations) between coverage of a particular motif in individual ChIP-seq samples and the mean coverage calculated for this motif in all samples. Thick black lines represent the cluster means. (**B**) Distribution of motifs from the four clusters in the PAO1161 genome. Inset represents an example of motif coverage profiles for selected motifs from cluster 2 and 4 in ChIP-seq samples derived from strains/indicated growth conditions.

The comparison of the coverage with reads around the half-*parS* GTTCCAC motifs in the genome showed limited preference for individual half-*parS* motif binding as motifs showing high coverage in one sample were also among the motifs with relatively high coverage in other samples (Figure 3E). As expected, the analysis demonstrated higher median coverage signal in samples derived from stationary cultures of the WT strain grown in rich medium in comparison to samples derived from fast-dividing cells (Figure 3E). To quantitatively assess the extent of ParB coverage enhancement of individual motifs in various samples obtained for cells grown on LB, the ChIP-seq coverage signal for individual motifs was extracted and normalized, and the principal coverage patterns in ChIP-seq samples for individual motifs were identified by k-means clustering (Figure 4A). 

Variation was observed dominantly in samples derived from logarithmic phase of growth. Cluster 3 contained motifs mapping to the region of the genome in the vicinity of *parS*s (Figure 4B), in which impaired ParB spreading in samples from the stationary phase of growth of the WT strain and samples of *parS1*–*4* mutant was observed (Figure 2A). For remaining clusters 1, 2 and 4, coverage was elevated in the WT samples derived from the stationary phase of growth, confirming change in the ParB distribution. For cluster 2, normalized coverage was elevated in samples of *parS1*–*4* from the logarithmic phase of growth (Figure 4B, see inset for an example). Motifs from this cluster were located in the one third of the genome around *oriC* (Figure 4B), which might suggest their role in the actively dividing cells of the mutant deprived of *parS1* to *parS4* sites to increase the local ParB concentration in this region; however, the possibility that this observed increase is a consequence of uneven *ori*/*ter* ratio relative to remaining samples could not be ruled out. The analysis of the relationship between half-*parS* coverage with ParB ChIP-seq reads and genome region GC content, motif localization on particular genome strand, and the extent of transcription in the region harboring the motif or gene expression changes as the cells were entering stationary phase did not reveal relations with the extent of individual motif coverage (Figure S3A–D); hence, the factors dictating the degree of ParB recruitment to individual half-*parS*s are currently unknown. Overall, these data indicate that the general extent of ParB binding to half-*parS*s could be affected by the environmental conditions, but the preference for binding to individual half-*parS* is not altered under the conditions tested.

### 2.3. Mutations in the Conserved Arginine Patch and C-Terminal Dimerization Domain of Pae ParB Impair Its Spreading Ability but Not Binding to Half-parSs

**Figure 5:**
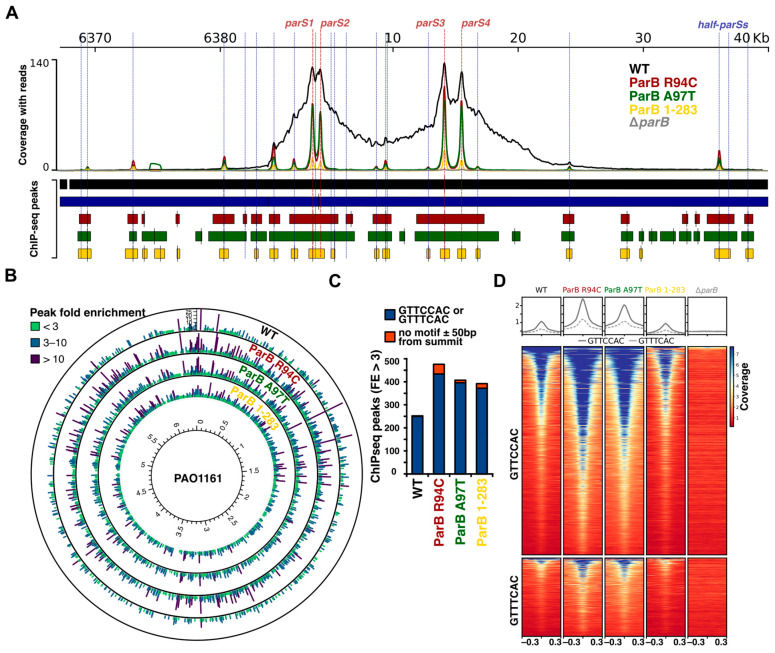
Effects of mutations in the arginine rich patch of ParB and deletion of the C-terminus on ParB interactions with DNA, assessed using ChIP-seq. (**A**) Coverage of regions encompassing the *parS1–parS4* in the PAO1161 genome in the ChIP-seq samples for the indicated strains. The histograms show normalized coverage with reads (averaged for two biological replicates). (**B**) Distribution of ChIP-seq peaks for the analyzed strains in the genome. Bar height and colors indicate peak fold enrichment (FE). (**C**) Number of peaks with and without GTTCCAC or GTTTCAC motifs ± 50 bp from the summit. The analysis was performed only for peaks with FE > 3. (**D**) Read coverage around GTTCCAC and GTTTCAC motifs in the PAO1161 genome in the ParB ChIP-seq samples of different strains. Individual lines in the heatmap represent normalized read counts for regions ±300 bp around a motif. The data were sorted in descending order of median coverage value and represent averaged value for two biological replicates. Plots indicate median coverage over the analyzed region for the two motifs.

The presence of narrow ChIP-peaks at *parS1–parS4* in WT cells in the samples from the stationary phase culture suggested diminishing ability of ParB to spread under these conditions. A previous study indicated that R94C or A97T substitution or removal of seven C-terminal amino acids (ParB 1-283) yields ParB variants unable to spread and silence test genes with the *parS* sequence placed upstream of their promoters [42]. The ParB R94 and A97 amino acids are located in the conserved GxxRxxA motif of helix 3, which was recently demonstrated to form a CTP-binding pocket in ParB homologues [12,13]. The *Pae* ParB R94C and A97T variants were defective in the polymerization in vitro (glutaraldehyde cross-linking) but still dimerized and bound *parS* efficiently in the EMSA assays [42]. The truncated ParB 1-283 variant was monomeric and highly impaired in DNA binding, at least in the EMSA assays [43]. Cultures of PAO1161 mutants expressing these variants contained a similar number of the anucleate cells as *parB* deficient strain, and the diffused ParB signal was observed in the immunofluorescence assays [42,43]. To analyze the interactions of these ParB variants with the genome, we performed ParB ChIP-seq experiments for cells grown exponentially in LB at 37 °C. The ParB R94C and ParB A97T bound efficiently to *parS1* to *parS4* sequences, but, in contrast to the WT ParB in the parental strain, the peaks formed in the mutants were narrow, confirming defective spreading (Figure 5A and Figure S4A). The dimerization-impaired truncated ParB 1-283 was able to bind specifically to DNA; however, the enrichment at *parS1* to *parS4* was around 10-fold lower than for WT ParB. For all three mutants, additional ParB bound regions were still present (Figure 5B). The vast majority of these ChIP-seq peaks contained half-*parS* motifs in proximity to the peak summits (Figure 5C), and analysis of the median read coverage around the half-*parS* motifs (GTTCCAC and GTTTCAC) confirmed the ParB binding to these two sequences (Figure 5D). These data indicate that ParB binding to half-*parS*s is not disturbed by R94C and A97T substitutions in ParB (likely impairing CTP binding/metabolism) but is also not determined by the ability of ParB to form dimers.

### 2.4. Effect of Half-parSs Removal on ParB Genome Distribution and Gene Expression

**Figure 6:**
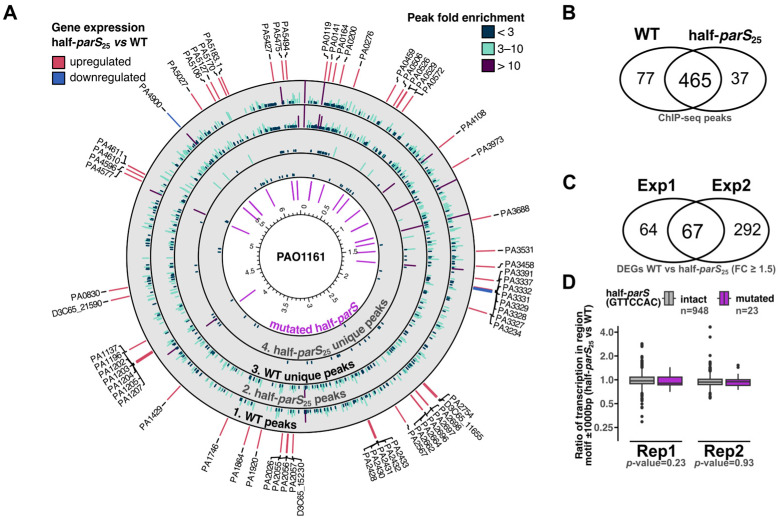
ParB binding to half-*parS* plays an indirect role in gene expression regulation in *P. aeruginosa*. (**A**) Circular plot indicating genomic localization of peaks identified in WT and half-*parS*_25_ strains (tracks 1 and 2) and peaks identified only in one of the strains (track 3 and 4). The positions of half-*parS* motifs modified in half-*parS*_25_ strain are colored with violet. The outer ring indicates 67 genes identified as differentially expressed in both RNA-seq experiments, colored according to the expression change trend. Labels indicate PAO1 gene IDs, or PAO1161 IDs (D3C65_), for genes annotated only in the PAO1161 genome. (**B**) Comparison of ChIP-seq peaks identified in WT and half-*parS*_25_ strains. (**C**) Comparison of sets of differentially expressed genes identified in two independent RNA-seq analyses. (**D**) Effect of the half-*parS* mutations on the transcription of surrounding genome fragments. Reads mapping to genome fragments ± 1000 bp from GTTCCAC motifs were counted in RNA-seq sample, and normalized for the total read count, and the ratio between WT and half-*parS*_25_ samples was calculated. Dots indicate outliers. Statistical significance between the two groups was evaluated using the Wilcoxon rank sum test.

The previous [31,32] and presented here ParB ChIP-seq analyses (Figure 1C and Figure 5B) indicated the existence of a set of genome regions, centered around half-*parS*s, reproducibly showing high fold enrichments. To assess the role of ParB binding to these particular sites, we have constructed the PAO1161 half-*parS*_25_ strain harboring nucleotide changes in 23 GTTCCAC and 2 GTTTCAC motifs, in a total of 18 loci, in the vicinity of the summits of ParB ChIP-seq peaks showing the highest FE (Figure 6A, inner ring, and Table S2).

Whole genome sequencing confirmed the introduction of mutations in the selected half-*parS*s; however, detailed analysis also indicated four nonsynonymous mutations (Table S2). The growth rate of the half-*parS*_25_ strain was not altered (Figure S5A), and the cultures of this strain did not exhibit elevated content of anucleate cells (Figure S5B). The ParB ChIP-seq analysis of half-*parS*_25_ strain showed that the introduced mutations abolished ParB binding in the 18 regions (Figure 6A), confirming that indeed half-*parS*s present in these regions are targets for ParB. The majority of ChIP-seq peaks were identified in the same genome regions in half-*parS*_25_ strain as in the WT (Figure 6B), and the differentiating peaks observed outside the mutated regions were mostly showing low FE (Figure 6A,B). Notably, analysis of the coverage with ChIP-seq reads of the genome region around *parS*s showed minor, but statistically significant enrichment of ParB in the region 10 kb downstream of *parS4* and 5 kb upstream of *parS1*, with the lower occupancy of the region between *parS2* and *parS3* (Figure S4B,C). This suggests that mutations in the selected, distantly located half-*parS*s may influence ParB interactions with the *parS1*–*parS4* region. 

The effects of changes introduced in the pattern of ParB binding to the genome of half-*parS*_25_ mutant on the transcriptome were analyzed by RNA-seq (Table S3)

Two independent analyses indicated a set of 67 genes differentially expressed in half-*parS*_25_ mutant, relative to the parental strain (Figure 6C, Table S3). For these genes, the observed trend of gene expression changes was the same in both RNA-seq analyses, except *PA3234*, and minor variation was observed between individual replicates (Figure S6). An inspection of the gene expression changes in regions adjacent to mutated half-*parS*s showed altered expression of only two genes (Table S3). Altering the GTTCCAC sequence in the region preceding *PA4900* reduced the expression of this gene, and modification of the same sequence in the *PA5127* resulted in its elevated expression. A global analysis of the transcription around mutated GTTCCAC motifs in comparison with remaining intact motifs did not reveal significant differences, suggesting no dominant effect of ParB binding to half-*parS*s on the local transcription (Figure 6D). The majority of genes identified as differentially expressed in half-*parS*_25_ mutant in comparison to the parental strain were upregulated and located at the distance from the introduced genome modifications (Table S3, Figure 6A). Of the 67 genes, 17 were classified as dependent on the bistable regulator BexR (PA2432), based on the comparison with its previously described regulon [44,45]. The *bexR* and several genes identified here were previously identified as differentially expressed in response to a mild ParB overproduction in *Pae* [46], suggesting a response of this regulator to alterations in ParAB–*parS* system functioning. The genes with changed expression in half-*parS*_25_ also encompassed multiple transcriptional regulators, including PA3458 [41], PA3973 [47], DdaR (PA1196) [48] and several other uncharacterized transcriptional regulators (Table S3). Overall, these data indicate the indirect role of ParB binding to half-*parS* sites in regulation of gene expression.

## 3. Discussion

The ParB–*parS* interactions, followed by spreading of the protein on adjacent DNA and formation of nucleoprotein complexes are universal features of these DNA segregation systems [11,49]. For *B. subtilis* ParB (Spo0J), non-specific DNA binding by the C-terminal part of the protein was also demonstrated [50,51]. Recently, ChIP-seq analyses of DNA binding for *Pae* ParB [32] as well as for ParB homologues from various bacterial species, upon their heterologous expression in *E*. *coli*, showed that certain ParBs exhibit significant binding to half-*parS* sequences, corresponding to one arm of the *parS* palindrome [5]. This work aims at elucidating the role of ParB binding to half-*parS* sites in *Pae*.

Previous analyses indicated different extent of half-*parS*s occupancy in cells overproducing ParB or in cells with mutated *parS*s [32]. Here, we analyzed the distribution of *Pae* ParB on the genomes in the fast- and slow-growing WT cells from exponential and stationary phases of culture growth. ChIP-seq experiments showed significant differences in the ParB binding patterns between two phases of culture growth in rich medium (Figure 1C). In fast dividing cells (in which the replication and division processes are uncoupled), the majority of ParB occupies *parS1* to *parS4* sequences and spreads around them, forming the large nucleoprotein complex, and a small fraction of ParB is distributed to half-*parS*s scattered in the genome (Figure 2A and Figure 3D). In contrast, in cells from the corresponding stationary cultures, the majority of recovered ParB-bound DNA encompassed half-*parS* motifs, and low recovery was observed for genome regions around *parS*s (Figure 2A), suggesting limited ParB spreading. This was in agreement with the microscopic analysis showing a gradual decrease in detectable ParB foci as the cells entered the stationary phase (Figure 2C,D). We speculate that when unrestricted growth in the rich medium can no longer be maintained due to depletion of nutrients, the cell density and/or composition of the spent medium, the culture enters the stationary phase, and the need to preserve intact ParB–*parS* complexes declines. Since it was demonstrated that the formation of ParB–*parS* complexes depends on the availability of CTP [12,13,14,15,16], one of the signals to disperse ParB–*parS* complexes may be the diminished CTP pool. Intriguingly, in the search for *Pae* ParB partners, we have discovered multiple proteins, including those with putative metabolic activities, among them *Pae* CTP-synthetase PyrG (PA3637) [34].

The dispersion of ParB–*parS* complexes in the cells grown on rich medium upon entering the stationary phase was accompanied by ParB enrichment on half-*parS* sites (Figure 3D,E). The biological role of the ParB shifts from *parS*s to half-*parS* sites during transition from uncoupled to coupled growth, characterized by limitation of replication initiation events, and prolongation of generation time remains highly speculative. The hypothetical significance of such relocation could be (i) storage of ParB molecules, rendering them instantly available for complex formation on *parS*s upon return to conditions of unrestricted growth; (ii) ParB protection against proteolytic degradation; or (iii) a role in altering DNA topology.

Notably, when ChIP-seq analyses were conducted with slow growing WT cells (minimal medium), in which the replication and cell division processes are constantly coupled, the ParB–*parS1–parS4* nucleoprotein complexes were present in the cells from logarithmic as well as stationary phases of growth (Figure 2B,E,F). The number of ParB-enriched half-*parS* sites in the ChIP-seq analysis did not differ between samples from the various phases of slow growth, confirming that the transition from fast uncoupled to sustained coupled growth observed in the case of cells grown in rich medium induces changes in ParB distribution on the genome. The presence of ParB–*parS* complexes in non-dividing cells, in which they are not engaged in DNA segregation, may suggest their involvement in another important cellular function in, e.g., anchoring of the genome in the cells [5]. As the ParB–*parS* complexes fulfill multiple hallmark criteria for liquid–liquid phase separation [52], it is tempting to speculate about their function in metabolic compartmentalization within bacterial cells; however, further studies are needed to address this possibility.

Our ChIP-seq analysis of strains producing ParB R94C and ParB A97T demonstrated the lack of the ParB–*parS1*–*parS4* complex near *oriC* (Figure 5A). Significantly, both these ParB variants did not lose the ability to bind *parS*s and half-*parS*s. Previously, we have shown that the truncated ParB 1-283 variant, with the impaired dimerization domain, was defective in all ParB biological roles in *Pae* [43]. The ChIP-seq experiments presented here confirmed that this variant was defective in spreading (Figure 5A). Surprisingly, it was still able to bind to *parS*s and half-*parS*s although with lower enrichment, suggesting that binding to DNA is not strictly dependent on ParB dimer formation. Recent studies shed light on the structural rearrangements within ParB molecules upon *parS* binding and diffusing to the surrounding DNA. In DNA unbound apo form, ParB adopts an autoinhibited conformation due to interactions between N-terminal and central (M-) domains of a single molecule [53]. Upon *parS* binding, the N-domain is untethered from the M-domain and becomes available to interact with the N-domain of the second molecule within the ParB dimer. This interaction (N–N engagement) closes the molecule, which is followed by M–M domain engagement, releasing the DNA molecule into a chamber formed by flexible linkers between M- and C-terminal dimerization domains, rendering the molecule DNA bound in a ring-like form [12,13,15,53]. The experiments supporting these observations were conducted with ParB from *B. subtilis* and *Caulobacter crescentus*, for which the property to bind half-*parS* sites was not observed (*B. subtilis*) or was relatively weak, respectively [5]. The observed diversity in half-*parS* binding might suggest different requirements of different ParB homologues for effective DNA binding, dictated, e.g., by the N–M engagement.

Removal of 25 half-*parS*s, located in 18 loci showing highest ParB enrichment in ChIP-seq, did not change the phenotype of half-*parS*_25_ relative to the parental strain. Despite removal of only a fraction of the ParB recognized motifs and performing the experiments on unsynchronized cell populations, some interesting observations have been made. Firstly, introduced modifications not only aborted ParB binding to these 25 sites scattered around the genome but also slightly altered the ParB–*parS* complex formation at *oriC* region (Figure S4C). Secondly, the transcriptomic analysis demonstrated the upregulation of 67 genes mostly in genome regions not adjacent to the modified loci (Figure 6A). It was previously suggested that global changes in the transcriptome of *parB* deletion mutant may be caused by indirect interactions of ParB with various partners or topological changes [33,34]. So far, the direct influence of *Pae* ParB on gene expression has been demonstrated only for ParB binding to the intergenic *parS3* and *parS4* [46].

To summarize, the role of half-*parS*s in the biology of *Pae* remains enigmatic. At present, they seem most likely to act as the reservoir of ParB in the cells. Under conditions of declining demand for the rapid chromosome segregation, ParB complexes are dispersed, and ParB shifts to multiple half-*parS* sites serving as the storage places, which overall supports the adaptation to changing environmental conditions. Additionally, future studies will elucidate their role in topology of the *Pae* chromosome.

## 4. Materials and Methods

### 4.1. Strains and Growth Conditions

Bacterial strains used in this study are listed in Table 1. *P. aeruginosa* strains, derivatives of PAO1161 [37], were grown at 37 °C or 30 °C, in LB (1% Bacto tryptone, 0.5% yeast extract, 0.5% NaCl, BD Difco, Franklin Lakes, NJ, USA) or in M9 medium [54], containing 0.5% glucose as a carbon source and supplemented with and 100 μg mL^−1^ leucine. Overnight cultures in LB medium were diluted 1:200 (LB) or 1:100 (M9), in a total volume of 20 mL in a 100 mL flask closed with a cotton plug. Culture growth was monitored by optical density measurements at 600 nm or by performing serial dilutions, plating on LB agar and counting of colonies after 20 h of growth at 37 °C. Iterative allele exchange to construct PAO1161 half-*parS*_25_ was conducted with the use of suicide vector pAKE600 [55] using the two-step method involving selection of transconjugants on LB medium with 300 μg mL^−1^ carbenicillin and 300 μg mL^−1^ rifampicin (used for donor counterselection), and selection of colonies after vector removal by culturing the cells in LB medium with 10% sucrose, as described [34]. The construction of pAKE600 derivatives is described in Supplementary Table S2. Introduction of mutations was confirmed by analysis of sequencing data generated in this study using Breseq v0.36.0 [56] relative to the PAO1161 reference genome (accession number CP032126 [37]).

### 4.2. ChIP-seq

ChIP was performed essentially as previously described [32,59]. Briefly the cells were fixed with 1% formaldehyde, and chromatin was isolated and sonicated to obtain fragments shorter than 300 bp and incubated with rabbit polyclonal anti-ParB antibodies, affinity purified on Affi-Gel 10 (Bio-Rad, Hercules, CA, USA). After antibody recovery with use of magnetic beads, the bound DNA was de-crosslinked, purified and used for library preparation with a Qiagen QIAseq Ultralow Input Library Kit and sequenced on an Illumina Novaseq 6000. Fastp v0.22.0 [60] quality-controlled reads were mapped to the *P. aeruginosa* PAO1161 genome (accession number CP032126 [37]) using Bowtie2 v2.2.5 [61]. Coverage data normalized using reads per genome content (RPGC) were generated without smoothing using the bamCoverage function from the deepTools package v3.5.1 [62], for individual nucleotides (data used for heatmaps and motif coverage analyses, Figure 4) or for 100 bp bins (data used for visualization of coverage with reads). Peaks were called using combined data for replicates and corresponding ∆*parB* data using MACS2 v2.2.6, with default settings for paired end data [63]. Visualization of coverage data and peak ranges was performed with pyGenomeTracks v3.8 [64]. All statistical analyses were performed using R Statistical Software (v4.2.2; [65]). Circular plots were generated within R using the circlize package v.0.4.15 [66]. Venn diagrams for ChIP-seq peak datasets were generated using ChIPpeakAnno v3.32.0 [67], with connectedPeaks set to ”merge”. Calculation of the number of reads mapping to *parS* and half-*parS* containing genome regions in ChIP samples was performed using the plotEnrichement function from deepTools [62], with the remaining parts of the genome used to calculate the theoretical coverage background, proportionally subtracted from the obtained counts (Figure 3D). Visualization of the coverage with ChIP-seq reads around half-*parS*s (GTTCCAC and GTTTCAC motifs) on heatmaps was conducted using computeMatrix and plotHeatmap functions from deepTools. Correlation between peak sets was analyzed using the dba.plotHeatmap function of Diffbind package v3.8.4 [68]. Differential ParB binding in the data of half-*parS*_25_ strain relative to the parental strain was analyzed in 200 bp genomic bins using the dba.report function of Diffbind [68], with the option to include all sites in the report and the WT input sample as a control, similar to a previous description [32].

### 4.3. RNA-seq

Total RNA was isolated from 2 mL of cultures growing in LB medium at 37 °C, harvested at optical density 0.4 to 0.5. Three independent replicates of each strain were analyzed, and the whole analysis was repeated twice. Cells were fixed with RNAprotect Bacteria Reagent (Qiagen, Hilden, Germany), and RNA was isolated with an RNeasy mini-kit (Qiagen) according to the manufacturer’s protocol. Total RNA was digested with DNase (TURBO DNA-free Kit, Ambion, Vilnius, Lithuania), and absence of genomic DNA was confirmed with PCR. Subsequently, rRNA was depleted using Ribo-Zero rRNA Removal Kit for bacteria (MRZMB126, Illumina, San Diego, CA, USA), and libraries were prepared using a NEBNext Ultra Directional RNA Library Prep Kit for Illumina and sequenced. Data analysis was conducted as described previously [40]. Briefly, reads were quality controlled using fastp and mapped to the PAO1161 genome using Bowtie2. FeatureCounts v2.0.3 [69] was used to count the number of reads mapping to individual genes. Identification of differentially expressed genes was conducted using edgeR ver 3.40.2 [70], with a fold change cut-off of 1.5 and an FDR-adjusted *p*-value of <0.05.

### 4.4. Fluorescence Microscopy and Image Analysis

Overnight cultures of PAO1161 harboring pAKW1125 (*tacp*–*YFP*–*ParB*) were grown overnight in LB or M9 medium with chloramphenicol added at a concentration of 75 µg mL^−1^. After dilution to the fresh media without antibiotics, samples were collected in time intervals, and cells were fixed with formaldehyde/glutaraldehyde mixture essentially as described before [34]. Images were captured using a Zeiss Imager M2 equipped with a 100 × 1.30 NA Plan-Neofluar objective, a Zeiss AxioCam MRc5 camera, using AxioVision (AxioVs40 V 4.8.2.0, Carl Zeiss MicroImaging GmbH, Munich, Germany) software. Images were processed using ImageJ, and foci detection was performed using MicrobeJ ver 5.13m [71], as described [34]. Since the images obtained at different time points varied significantly in fluorescence background within the cells (possibly due to autofluorescence), the threshold for foci intensity detection in MicrobeJ was adjusted in individual sets of images, and proper selection was validated by manually counting foci in at least 100 cells.

## Figures and Tables

**Table 1:** Bacterial strains used in this study.

Strain Name	Description/Relevant Genotype	Origin
***Escherichia coli***		
DH5α	F^−^ Φ80*lacZΔM15 Δ(lacZYA-argF) U169 recA1 endA1 hsdR17* (r_K_–, m_K_+) *phoA supE44 λ thi-1 gyrA96 relA*	[57]
S17-1	*pro* Δ*hsdR hsdM^+^ recA* Tp^R^ Sm^R^ ΩRP4-*Tc*::*Mu Kn*::*Tn7*	[58]
***Pseudomonas aeruginosa***		
PAO1161 (WT)	Rif^R^, leu-, PAO1 derivative	[28,37]
∆*parB*	PAO1161 with *parB* deletion	[34]
*parS1*–*4*	PAO1161 with mutations in *parS1*, *parS2*, *parS3* and *parS4* impairing ParB binding; termed *parS*mut15 in the original paper	[30]
*parB94*	PAO1161 producing ParB R94C, non-spreading variant	[42]
*parB97*	PAO1161 producing ParB T97A, non-spreading variant	[42]
*parB* Δ*284-290*	PAO1161 producing truncated ParB 1-283 variant	[43]
half-*parS*_25_	PAO1161 with 25 half-*parS* sites modified in 18 loci	this work

## Data Availability

Raw sequencing data were deposited in the NCBI’s Gene Expression Omnibus (GEO) database (http://www.ncbi.nlm.nih.gov/geo/) under accession number GSE233379 (accessed on 19 May 2023).

## Supplementary Materials

The following supporting information can be downloaded at: https://www.mdpi.com/article/10.3390/ijms241512517/s1.
