# Supplementary material for: Robust ParB Binding to Half-parS Sites in Pseudomonas aeruginosa—A Mechanism for Retaining ParB on the Nucleoid?
Source: Int J Mol Sci. 2023 Aug 7;24(15):12517. doi: 10.3390/ijms241512517 (PMC10419367; doi:10.3390/ijms241512517)
Supplement: Supplementary file 1 [file ijms-24-12517-s001.zip › Suplemenatry Figures.pdf]

SUPPLEMENTARY FIGURES

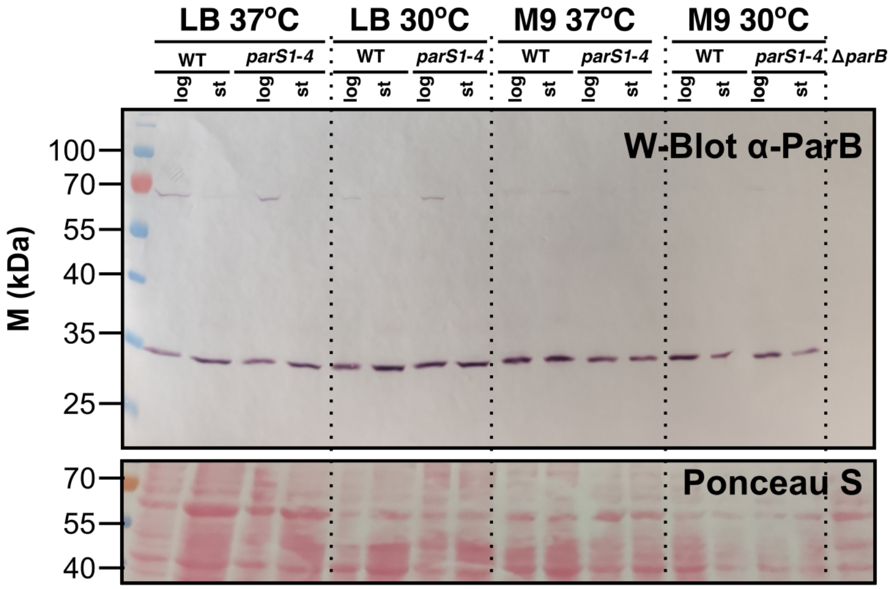

Figure S1. Western blot analysis of ParB level in cleared lysates obtained from *P. aeruginosa* cells grown in the indicated medium / temperature and harvested at two time points. Samples containing lysate volumes corresponding to 20 µg of total DNA were boiled, subjected to SDS-PAGE, and Western blot analysis with the use of anti-ParB antibodies. Ponceau S stained membrane is shown below.

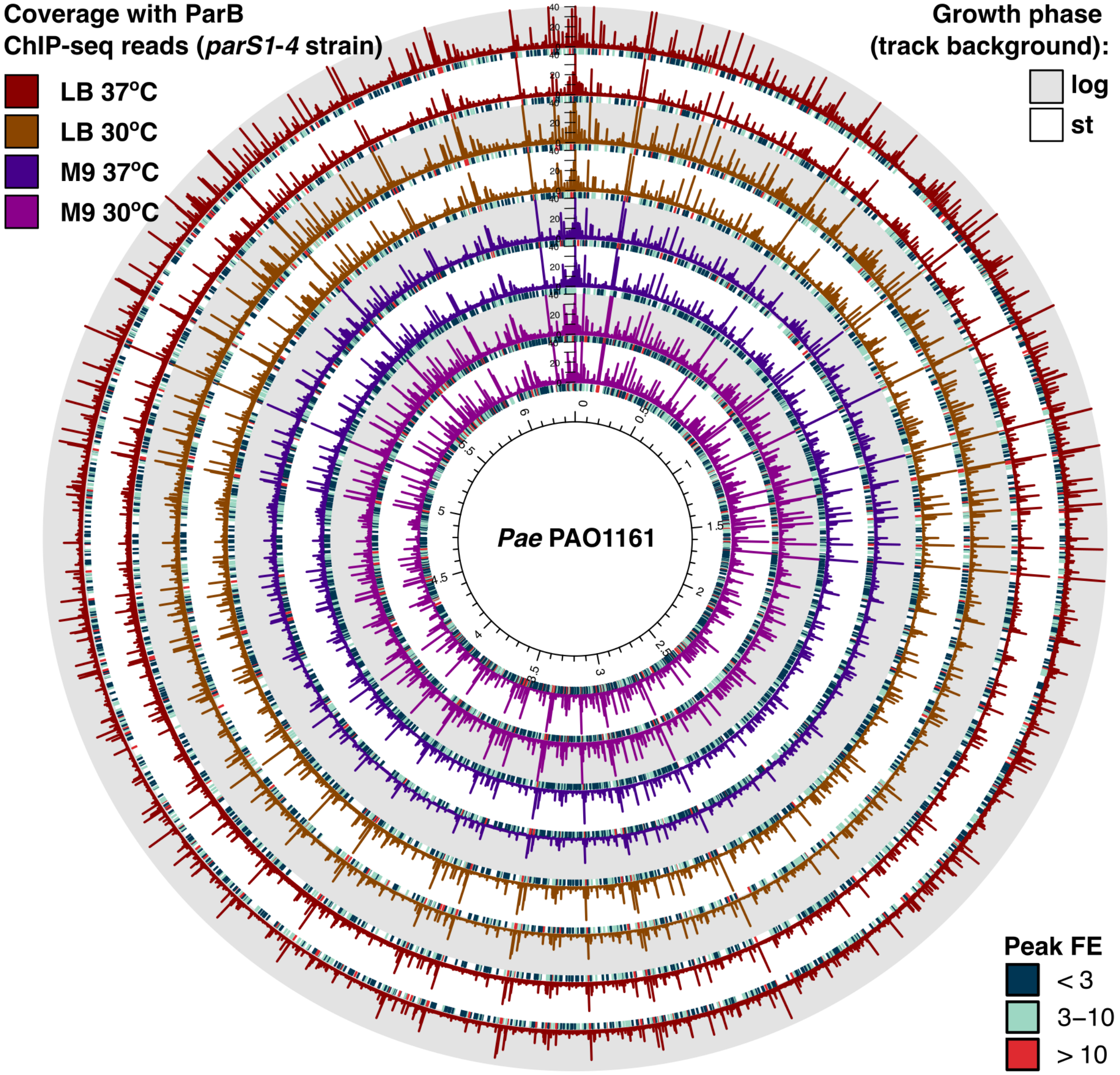

Figure S2 ParB binding pattern to *P. aeruginosa* in *parS1-4* cells under various culturing conditions. *P. aeruginosa parS1-4* strain was grown in LB or M9 medium supplemented with 0.5% glucose and 100 µg ml^{-1} leucine, at 30 or 37°C. Cells were collected at two stages of culture growth, and ParB ChIP-seq analysis was conducted. Track data represent normalised coverage of the genome with ChIP-seq reads (binned at 200 bp, and averaged for two ChIP-seq replicates). Colour of coverage track represents different growth conditions whereas track background indicates the phase of culture growth. Track height was set at 35% of max. coverage signal of the outer track. Short bars below each track represent the distribution of detected ChIP-seq peaks for these samples, coloured according to the peak fold enrichment (FE).

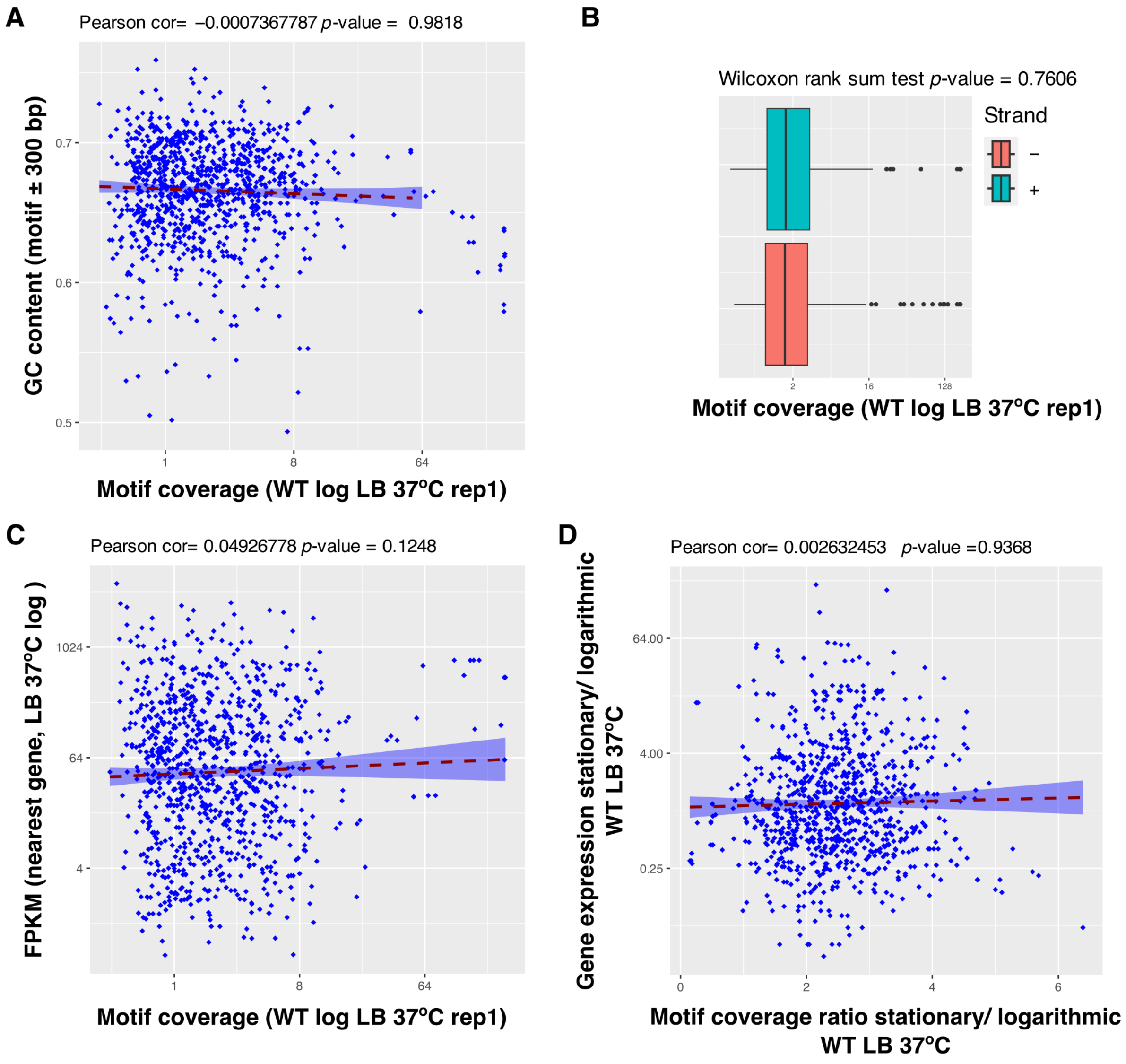

Figure S3 Relation between half-*parS* (GTTCCAC) coverage with ParB ChIP-seq reads and motif localization, genome region GC content and gene expression. Comparison of coverage data for 971 GTTCCAC motifs in samples derived from WT strain (LB 37°C, logarithmic phase of growth) with (**A**) GC content of genome fragment with the motif, (**B**) genome strand on which the motif is located and (**C**) the extent of gene expression of the nearest gene, analysed for the same strain/growth conditions (**D**) The relation between the motif coverage changes in samples derived from stationary vs logarithmic cultures with gene expression changes under the same conditions. Red lines indicate the smoothed conditional means. If the motif was intergenic, the nearest gene was used for analysis in (**C**) and (**D**). Data for gene expression changes between the phases were derived from [1]. Expression data for WT cells grown in LB at 37°C were taken for sample GSM6500804, from GSE211771 (http://www.ncbi.nlm.nih.gov/geo/ accessed 10.04.2023, reference [2]).

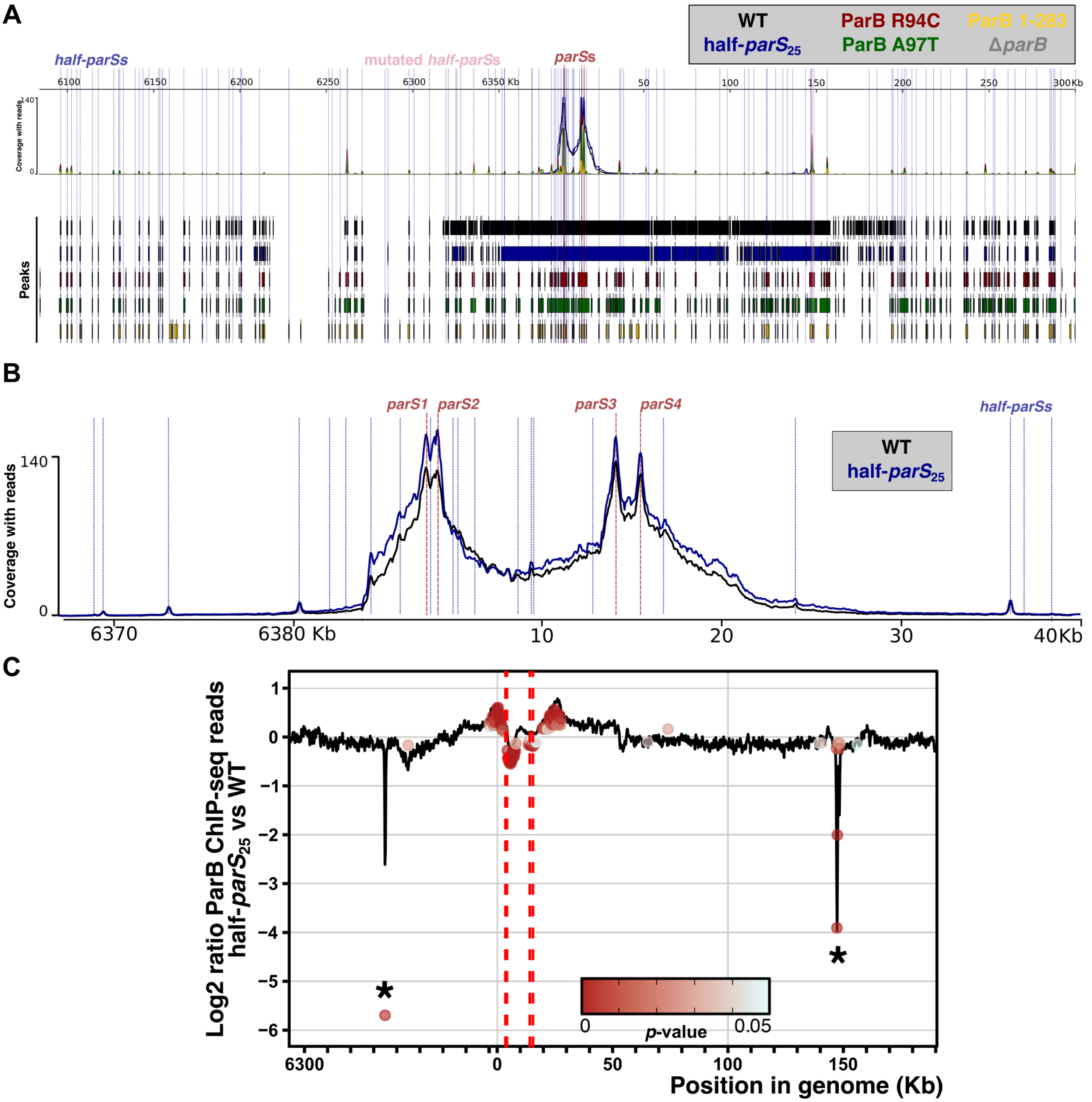

Figure S4. Coverage of the genome regions encompassing *parS1–parS4* with ChIP-seq reads samples derived from different strains. ParB ChIP-seq was performed using cells growing exponentially in LB medium at 37ºC and reads were mapped to the *Pae* PAO1161 genome. (**A**) Normalised coverage with reads for samples from PAO1161 WT, half-$parS_{25}$, Δ*parB* as well as strains expressing the indicated ParB variants defective in spreading. The bottom part indicates the ranges of detected ChIP-seq peaks, with vertical lines indicating peak summits (See Table S1). (**B**) Zoom in on the *parS1-4* region for WT and half-$parS_{25}$ strains. (**C**) Differential binding analysis of ParB binding to DNA in half-$parS_{25}$ strain *versus* WT. Analysis was performed in non-overlapping 200-bp windows covering the whole genome using Diffbind. The black line indicates smoothed ratio between reads mapping to each bin in ChIP-seq data, whereas bins with a significantly different coverage (FDR adjusted *p*-value <0.05) are indicated with circles. Asterisks indicate regions containing motifs mutated in half-$parS_{25}$ strain.

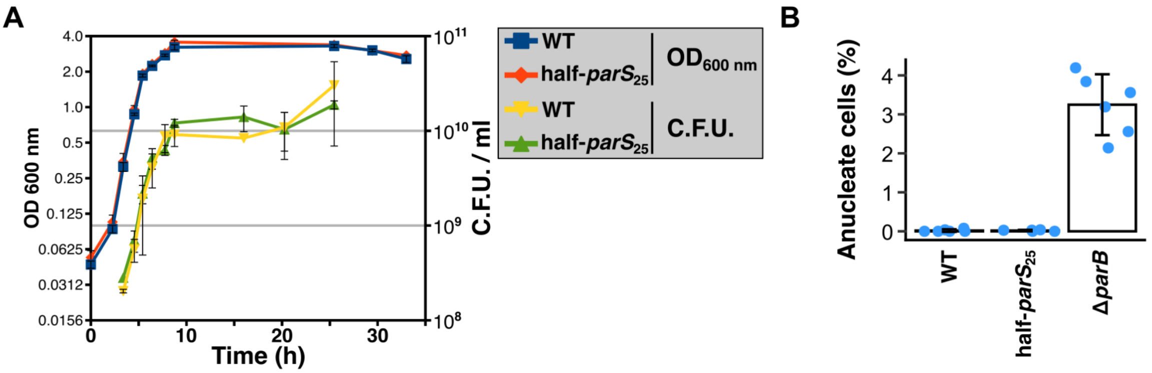

Figure S5. Phenotype characteristics of *P. aeruginosa* half-*parS*$_{25}$ strain. (**A**) Growth of PAO1161 WT and half-*parS*$_{25}$ strains in LB at 37°C. Data represent mean optical density at 600 nm and mean number of colony forming units (C.F.U.) per ml of culture ± SD from 3 cultures. (**B**) Percentage of anucleate cells in exponentially growing cultures (OD_{600} ~0.5) of indicated strains assessed by microscopic analysis after propidium iodide staining. Data represent mean ± SD, and blue dots represent the individual measurements of at least 1,000 cells.

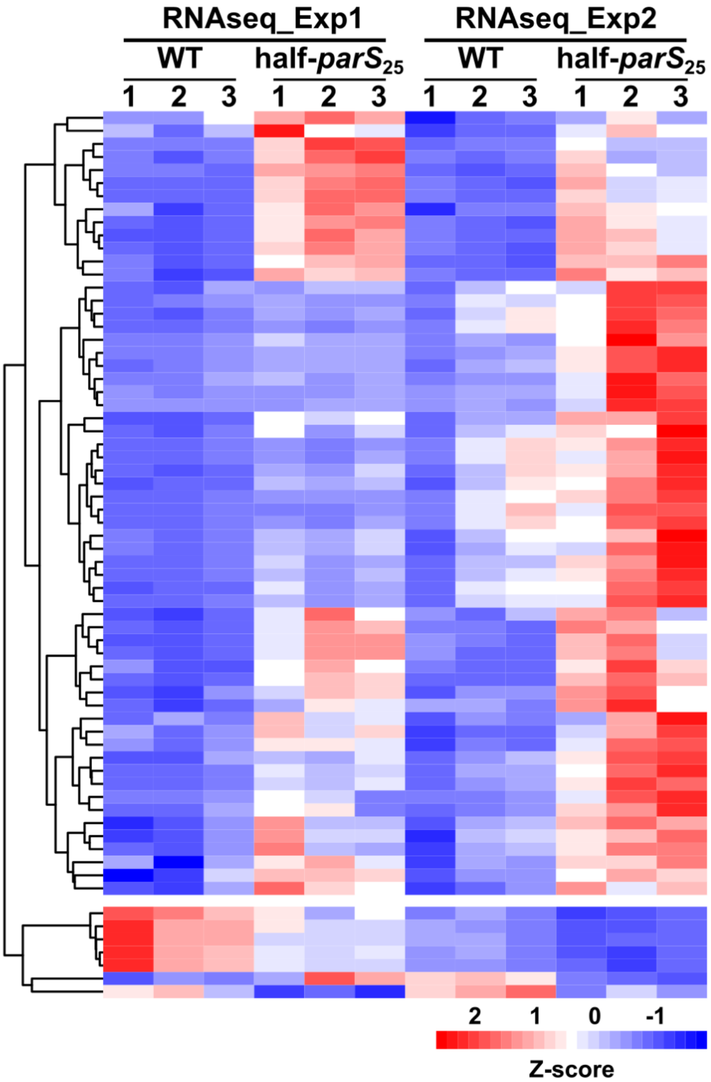

Gene_ID	Product	Mean FC (half-$parS_{25}$ vs WT) Exp1	Exp2
D3C65_21590	hypothetical protein	1.74	1.62
PA0830	metal-dependent hydrolase	1.87	2.01
PA0572	hypothetical protein	3.02	1.70
PA2698	hydrolase	3.28	1.79
PA2696	AraC family transcriptional regulator	2.67	2.11
PA2433	hypothetical protein	6.17	3.54
PA1202	hydrolase	9.66	4.43
PA2428	polyphosphate kinase 2	2.32	2.06
PA2432	bistable expression regulator, BexR	8.78	4.75
PA1204	NADPH-dependent oxidoreductase	3.04	2.25
PA1203	OsmC family peroxiredoxin	4.28	3.19
PA2057	TonB-dependent receptor, SppR	1.98	2.14
PA5127	probable rRNA methylase	1.81	1.57
PA2662	short-chain dehydrogenase	1.59	1.87
PA4610	copper transporter	1.59	2.80
PA1196	sigma-54-dependent Fis family transcriptional regulator, DdaR	1.68	1.95
PA4577	TraR/DksA family transcriptional regulator	1.56	1.97
PA3973	TetR/AcrR family transcriptional regulator	1.72	3.07
PA2026	bile acid:sodium symporter	2.82	7.58
PA1920	class III (anaerobic) ribonucleoside-triphosphate reductase subunit, Nrd	2.16	4.48
PA0529	MOSC domain-containing protein	1.69	5.38
PA0164	gamma-glutamyltransferase family protein	1.80	5.06
PA1137	oxidoreductase	1.69	7.52
PA0119	C4-dicarboxylate transporter 1	1.73	1.71
PA2567	sensor domain-containing phosphodiesterase	1.76	1.89
PA3337	ADP-glyceromanno-heptose 6-epimerase	1.93	1.97
PA2754	DUF883 domain-containing protein	1.50	1.65
PA3458	MarR family transcriptional regulator	1.58	1.68
PA5170	arginine:ornithine antiporter	2.28	2.15
PA0526	protein DnrP	1.63	2.18
PA1429	cation-transporting P-type ATPase	1.56	1.79
PA5427	alcohol dehydrogenase AdhP	1.76	1.83
PA0141	polyphosphate kinase 2	1.60	1.88
PA0459	AAA family protein disaggregase ClpG	1.86	2.45
PA4596	TetR/AcrR family transcriptional regulator, EsrC	2.71	3.22
PA5475	N-acetyltransferase	2.79	2.76
PA0200	DUF3079 domain-containing protein	1.81	1.75
PA5027	universal stress protein	2.32	2.64
PA2697	DUF1427 family protein	3.51	1.94
PA1205	pirin family protein	2.69	2.24
PA2431	FUSC family protein	4.07	2.88
PA2430	HlyD family secretion protein	4.32	2.91
PA0506	acyl-CoA dehydrogenase	1.56	1.59
PA2056	LysR family transcriptional regulator	2.87	2.85
PA2055	major facilitator superfamily (MFS) transporter	2.40	2.22
PA1207	glutathione-regulated potassium-efflux system protein KefB	1.58	1.57
PA3688	pilin assembly protein	1.69	1.88
PA3531	bacterioferritin	1.66	2.33
PA5106	formimidoylglutamate deiminase	1.82	2.73
PA0276	DUF962 domain-containing protein	1.71	2.28
PA4108	cyclic di-GMP phosphodiesterase	2.05	2.48
PA1746	Appr-1-p processing protein	3.46	3.99
PA3391	regulatory protein NosR	4.55	10.76
PA2664	flavohemoprotein	1.91	3.31
PA5183.1	carbon storage regulator, RsmN	1.69	1.64
PA4611	DUF465 domain-containing protein	2.05	1.80
PA5494	DUF2782 domain-containing protein	1.53	1.61
PA1864	TetR family transcriptional regulator	1.66	1.64
D3C65_11655	hypothetical protein	2.05	1.69
D3C65_15230	hypothetical protein	2.90	2.91
PA3332	nuclear transport factor 2 family protein	0.66	0.56
PA3328	FAD-dependent monooxygenase	0.64	0.60
PA3327	non-ribosomal peptide synthetase	0.59	0.61
PA3331	cytochrome P450	0.64	0.59
PA3329	hypothetical protein	0.62	0.56
PA3234	cation acetate symporter	1.76	0.61
PA4900	major facilitator superfamily (MFS) transporter	0.63	0.64

Figure S6. Gene expression changes in half-*parS*$_{25}$ strain. The RNA-seq was conducted with cells from LB medium at 37°C in the logarithmic stage of culture growth. Heat map indicates expression changes in two repeats of RNA-seq analysis, each with 3 biological replicates for each strain for 67 genes identified as differentially expressed in both analyses (Figure 6C).

REFERENCES:

1.Alpers, K.; Vatareck, E.; Gröbe, L.; Müsken, M.; Scharfe, M.; Häussler, S.; Tomasch, J. Transcriptome dynamics of *Pseudomonas aeruginosa* during transition from overlapping to non-overlapping cell cycles. *mSystems* **2023**, *8*, e0113022.

2.Kotecka K, Kawalek A, Modrzejewska-Balcerek M, Gawor J, Zuchniewicz K, Gromadka R, Bartosik AA. Functional Characterization of TetR-like Transcriptional Regulator PA3973 from *Pseudomonas aeruginosa*. *Int J Mol Sci*. **2022**, *23*, 14584
