# Supplementary material for: Robust ParB Binding to Half-parS Sites in Pseudomonas aeruginosa—A Mechanism for Retaining ParB on the Nucleoid?
Source: Int J Mol Sci. 2023 Aug 7;24(15):12517. doi: 10.3390/ijms241512517 (PMC10419367; doi:10.3390/ijms241512517)
Supplement: Supplementary file 1 [file ijms-24-12517-s001.zip › Suplemenatry Tables Captions.pdf]

SUPPLEMENTARY TABLES

Table S1 ParB ChIP-seq peaks identified in different samples. Peaks were identified using MACS2, in various samples using the data obtained for Δ*parB* strain as a control. Sheet name indicates the strain and growth conditions.

Table S2 Motifs modified in half-$parS_{25}$ strain. Peak numbers indicate those from Table 1 in Kawalek et al., NAR (2018), 46, 4592–4606. The construction of vectors for allele exchange involved PCR amplification of 2 fragments from PAO1161 genomic DNA using oligo pairs A/B or C/D, respectively, followed by their digestion with indicated enzymes and simultaneous ligation with EcoRI and BamHI digested pAKE600, unless stated otherwise. Sheet "Verification" contains nucleotide changes identified in the genome of half-$parS_{25}$ strain. The analysis was conducted Breseq using PAO1161 genome (accession number CP032126) as reference and NGS data generated in this study.

Table S3. Gene expression profiling of *P. aeruginosa* half-*parS*25 strain. The results of transcriptome analyses for all PAO1161 genes are presented in sheet "All_genes" whereas data for differentially expressed genes (FC>1.5 or FC <-1.5, FDR adjusted *p*-value <0.05) in at least one of the RNA-seq experiments are included in "DEGs" sheet.
